# Supplementary material for: Thermal and electrostatic tuning of surface phonon-polaritons in LaAlO3/SrTiO3 heterostructures
Source: Nat Commun. 2023 Nov 24;14:7686. doi: 10.1038/s41467-023-43464-z (PMC10673882; doi:10.1038/s41467-023-43464-z)
Supplement: Supplementary file 1 — Supplementary Information [file 41467_2023_43464_MOESM1_ESM.pdf]

Thermal and electrostatic tuning of surface phonon-polaritons in $LaAlO_3/SrTiO_3$ heterostructures

Yixi Zhou[1,2], Adrien Waelchli[1], Margherita Boselli[1], Iris Crassee[1], Adrien Bercher[1], Weiwei Luo[1,3], Jiahua Duan[4], J.L.M. van Mechelen[5], Dirk van der Marel[1], Jérémie Teyssier[1], Carl Willem Rischau[1], Lukas Korosec[1], Stefano Gariglio[1], Jean-Marc Triscone[1], and Alexey B. Kuzmenko[1*]

[1]Department of Quantum Matter Physics, University of Geneva, CH-1211 Geneva 4, Switzerland
[2]Beijing Key Laboratory of Nano-Photonics and Nano-Structure (NPNS), Department of Physics, Capital Normal University, Beijing 100048, China
[3]The Key Laboratory of Weak-Light Nonlinear Photonics, Ministry of Education, School of Physics and TEDA Applied Physics Institute, Nankai University, Tianjin 300457, China
[4]Department of Physics, University of Oviedo, Oviedo 33006, Spain
[5]Department of Electrical Engineering, Eindhoven University of Technology, 5600 MB Eindhoven, Netherlands

[*] Email : Alexey.Kuzmenko@unige.ch

Supplementary Information

S1. Illumination geometries

As mentioned in the main text, two illumination geometries are studied (Fig. S1). A thick sample was partly covered by a metal film (Au or Pt) with a thickness of 30 nm. Incident light (E_{in}) illuminates on two orthogonal metal edges (1 and 2) with its projection almost parallel to the edge 1 and nearly perpendicular to the edge 2. While the incident angle in the two cases is the same, the azimuthal angle on the edge 2 differs from the one on edge 1 by 90°. The edge-launched surface polaritons dominates with corresponding propagation angle φ_1 and φ_2 to the two edges.

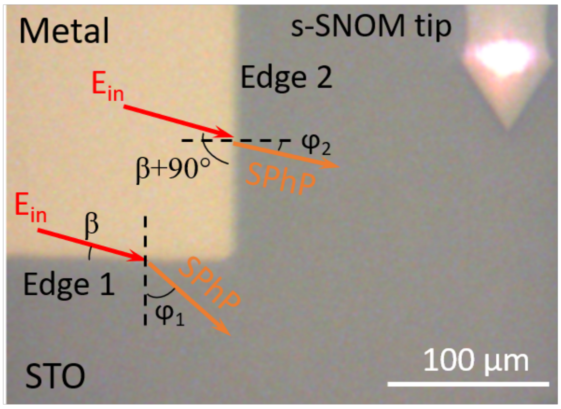

Figure S1. A photograph showing a bulk STO sample partly covered by metal and the cantilever of an AFM tip, made directly in the s-SNOM setup. Red arrows indicate the approximate direction of the illumination. Orange arrows represent the calculated direction of the propagation of the edge-launched SPhPs at 690 cm^{-1}. The actual s-SNOM scans were performed perpendicular to the edge at locations of

more than 200 μm from the corner (the dashed lines are shown close to the corner for the sake of the drawing compactness).

S2. SPhPs in STO launched by the metal edge 2

In Fig. S2a, we present the hyperspectral *x*-*ω* map of the s-SNOM amplitude s_2 obtained on the metal edge 2 on top of bulk STO at 15 K and normalized to the signal on metal $s_{2,\mathrm{met}}$. Below approximately 720 cm^{-1}, a series of dispersive fringes can be seen and persist at 140 μm away from the edge 2. We fit the nano-FTIR line profiles at different frequencies ω (a representative example at ω = 690 cm^{-1} is shown in Fig. S2b) using the damped-sine function as described in the main text and plot the extracted fringe spacing d_{exp} (red squares) and decay length L_{exp} (pink squares) as a function of *ω* (Fig. S2c and S2d). The fitted results based on Equation (1) of the main text ($\alpha = 23.3^{\circ}$, $\beta = 7.8^{\circ}+90^{\circ}$) agrees well with the d_{exp} and is 2 to 3 times larger than d_{exp} observed on the edge 1 (Fig. 1e in the main text).

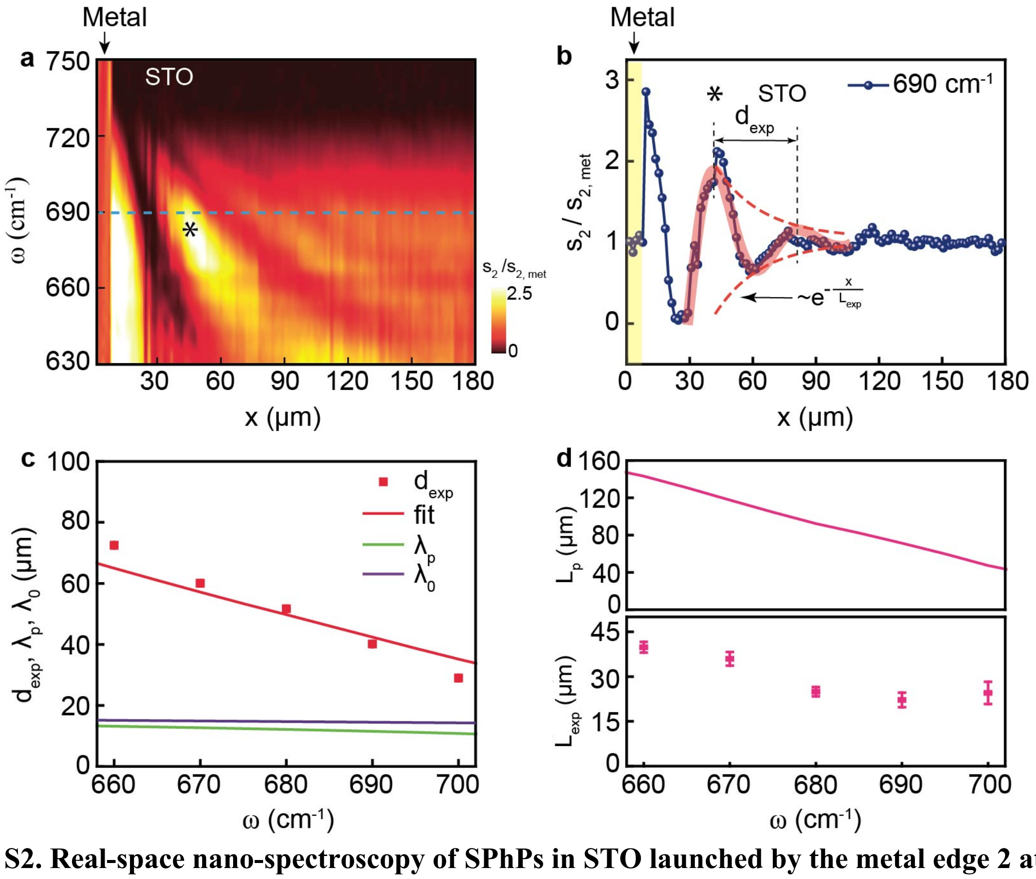

Figure S2. Real-space nano-spectroscopy of SPhPs in STO launched by the metal edge 2 at 15 K. **a**, Nano-FTIR line scans showing the metal-normalized near-field amplitude $S_2/S_{2,\mathrm{met}}$ as a function of the distance *x* between tip and metal. **b**, line profile (blue symbols) taken along the dashed line in **a** at 690 cm^{-1}. Solid line: a damped-sine function fit. **c,** Symbols: d_{exp} as a function of the wavenumber of incident light. Red solid line: the best fit when fitting parameters $\alpha = 23.3^{\circ}$, $\beta = 7.8^{\circ}+90^{\circ}$. Green and purple lines: the calculated SPhP wavelength λ_{p} and the incident light wavelength λ_0. **d**, Calculated propagation length L_{p} (upper panel) and experimental decay length L_{exp} (lower panel) as a function of the wavenumber.

S3. Calculation of the fringe spacing

Here we derive an expression for the SPhP fringe spacing in the s-SNOM profiles based on the optical phase arguments. We consider a general direction of the incident beam characterized by the incidence angle α and the azimuthal angle β (Fig. S3). For simplicity, we assume that only the photons scattered in the exactly opposite direction are collected by a remoted detector. Thus, we neglect a certain convergence of the incident beam and a finite acceptance angle for the scattered beam.

The fringe pattern appears due to the interference between the two scattering processes where the metal edge launched SPhP wave propagate to the tip and scattered into free space by the tip (path P1) and the one in which the photon is directly backscattered by the tip (path P2). We note that a third scattering process P3 exists, where the propagation of all waves is inverted with respect to path P1. However, the phase in this process is identical to the one in path P1, therefore we merge treat them as a single channel. As the SPhPs are weakly confined, we ignore the process where the polaritons propagate twice from the tip to the edge and back[1]. We also do not consider the photon back reflection from the edge since it is cancelled after the demodulation of the signal at the tip tapping frequency.

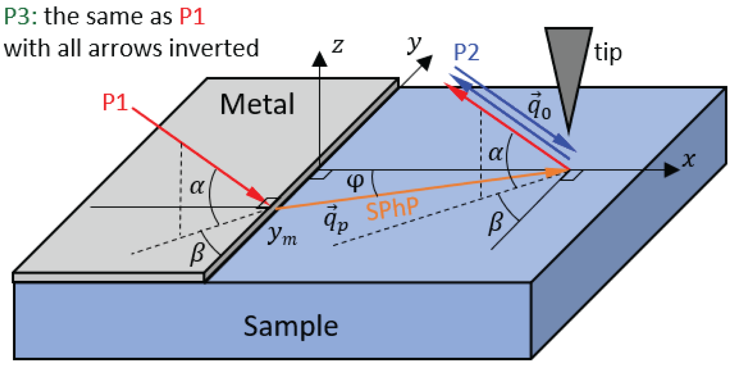

Figure S3. Illustration of the experimental set-up and various optical paths from which the photons can be collected

We assume that the tip is in the position $(x, y = 0)$ and we first consider a partial SPhP wave in the process P1 emitted from an arbitrary point $(x = 0, y)$. Based on geometrical arguments, one obtains the following expression for the phase difference between paths P1 and P2:

$$\Delta\Phi(x, y) = q_0 \cos\alpha\, (x \sin\beta + y \cos\beta) + q_{\mathrm{p}}\sqrt{x^2 + y^2} \quad , \qquad \text{(S1)}$$

where $q_0 = 2\pi/\lambda_0$ is the wavevector of incident light, $q_{\mathrm{p}} = 2\pi/\lambda_{\mathrm{p}}$ is the SPhP wavevector, α and β are the incident and azimuthal angle of the incident light, respectively.

In principle, one should sum up partial SPhP waves from all the edge points. To avoid the integration over y, one can apply the Fermat principle and consider only the point y_m on the edge corresponding to the shortest optical trajectory, where $\left.\frac{\partial\Delta\Phi(x,y)}{\partial y}\right|_{y=y_m} = 0$. Applying this condition results in the relation $q_0 \cos\alpha \cos\beta = q_{\mathrm{p}} \sin\varphi$, or

$$\lambda_{\rm p}\cos\alpha\cos\beta = \lambda_0\sin\varphi\ , \qquad \text{(S2)}$$

where $\varphi = \tan^{-1}(-y_m/x)$ is the angle of the SPhP propagation. Equation (S2) can be regarded as a surface-polariton analogue of the Snell's law. The phase difference between paths P2 and P1, where only the partial wave emanating from point y_m is considered, is

$$\Delta\Phi_m(x) = \Delta\Phi(x, y_m) = x\left[q_0(\cos\alpha\sin\beta - \tan\varphi\cos\beta) + q_{\rm p}/\cos\varphi\right]. \qquad \text{(S3)}$$

The fringe spacing along the x direction can be obtained by the equation: $\Delta\Phi_m(d) = 2\pi$:

$$d = 2\pi\cos\varphi\left[q_0(\cos\alpha\sin\beta\cos\varphi - \cos\beta\sin\varphi) + q_{\rm p}\right]^{-1}. \qquad \text{(S4)}$$

Considering (S2), we get:

$$d = 2\pi\cos\varphi\left[q_{\rm p} - q_0\cos\alpha\ \sin(\varphi-\beta)\right]^{-1}, \qquad \text{(S5)}$$

or, as in the main text:

$$d = \lambda_{\rm p}\cos\varphi\left[1 - (\lambda_{\rm p}/\lambda_0)\cos\alpha\ \sin(\varphi-\beta)\right]^{-1}. \qquad \text{(S6)}$$

To crosscheck this result with previous publications, we apply this general formula to two selected illumination geometries. In the 'perpendicular' geometry $\beta = \pi/2$, $\varphi = 0$ and $d = \frac{\lambda_p}{1-(\lambda_p/\lambda_0)\cos\alpha}$, as in Refs.[1, 2, 3]. In the 'parallel' geometry we have $\beta = 0$, $\varphi = \sin^{-1}\left(\frac{\lambda_p}{\lambda_0}\cos\alpha\right)$ and $d = \frac{\lambda_p\cos\varphi}{1-(\lambda_p/\lambda_0)\cos\alpha\cdot\sin\varphi}$, in agreement with Ref.[1] .

S4. Finite-dipole model of sample-tip interaction for the s-SNOM signal

In the paper we use a version of the finite dipole model (FDM)[4] adapted for multilayers[5]. We follow closely the original method, with, however, a few adaptations. Below we present the details of our numerical procedure.

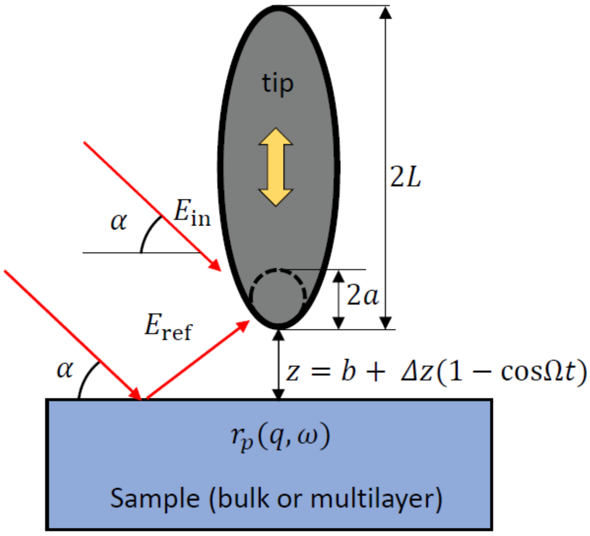

Figure S4. Description of the parameters used in the finite-dipole model.

The properties of the sample are fully characterized by its momentum- and frequency dependent reflection coefficient $r_p(q,\omega)$. It can be calculated for any bulk and multilayer

sample using the standard transfer-matrix formalism (an example is given in Ref.[6]). The tip in this model is approximated by an elongated ellipsoid with the half-length L and the apex radius a. The complex valued s-SNOM signal $\tilde{S}(z) = Se^{i\theta}$, which depends on the tip-sample distance z, is proportional to the illumination field E and the effective tip polarizability α_{eff} : $\tilde{S}(z) = \alpha_{\text{eff}}(z)E$ (the proportionality coefficient is unimportant, as it is cancelled in the normalization procedure, and we set it to 1). The illumination field is the sum of the directly incident field E_{in} and the field reflected from the sample E_{ref}=$E_{\text{in}}r_p(q_0 \cos\alpha, \omega)$:

$$E = E_{\text{in}}[1 + r_p(q_0 \cos\alpha, \omega)]. \quad \text{(S7)}$$

The effective tip polarizability is affected by the near-field sample-tip interaction and is given by the formula[5]:

$$\alpha_{\text{eff}} = \alpha_0 \left(1 + \frac{1}{2}\frac{\beta_0 f_0}{1-\beta_1 f_1}\right), \quad \text{(S8)}$$

where α_0 is the bare polarizability in the absence of the sample. Here $\beta_{0,1} = \frac{-\varphi_{0,1}^2}{\varphi'_{0,1}}$, where $\varphi_{0,1} = -\int_0^\infty r_p(q,\omega)e^{-2qZ_{0,1}}dq$ and $\varphi'_{0,1} = -\int_0^\infty r_p(q,\omega)qe^{-2qZ_{0,1}}dq$. Furthermore, $f_{0,1} = \left(g - \frac{a+z+X_{0,1}}{2L}\right)\frac{\ln\left(\frac{4L}{a+2z+2X_{0,1}}\right)}{\ln(4L/a)}$, where $Z_{0,1} = W_{0,1} + z$, $X_{0,1} = \frac{\varphi_{0,1}}{\varphi'_{0,1}} - Z_{0,1}$, $W_0 = \frac{1.31La}{L+a}$, $W_1 = 0.5a$ and $g = 0.7 + e^{i0.06}$.

To capture the q-dependence in $\varphi_{0,1}$ and $\varphi'_{0,1}$ accurately, the integral is performed using a logarithmically spaced grid in q. In this way, the variations of $r_p(q,\omega)$ for small q values are taken better into account, leading to an improved numerical precision and significantly reduced calculation time.

In the s-SNOM experiment, the tip is oscillating with the frequency Ω: $z(\tau) = b + \Delta z * (1 - \cos\tau)$: where b is the shortest distance to the sample, Δz is the tapping amplitude and $\tau = \Omega t$ is the tapping phase. The signal demodulated at n-th harmonics of Ω is calculated by the Fourier transform:

$$\tilde{s}_n = s_n e^{i\theta_n} = (2\pi)^{-1}\int_0^{2\pi} \tilde{S}[z(\tau)]e^{in\tau}d\tau. \quad \text{(S9)}$$

According to the experimental conditions, we used the values a = 60 nm, $2\Delta z$ = 90 nm, b = 0. In all simulation of adopted α = 23.3° according to the fits of the data on two orthogonal edges as described in the text. We set L to 320 nm and we checked that changing this parameter within reasonable limits does not affect any trends seen in the model spectra.

S5. Hyperspectral nano-imaging of SPhPs in LAO/STO

Figure S5 illustrates a hyperspectral x-ω map of SPhPs in LAO/STO heterostructures at 10 K, where the STO is covered by a LAO layer with 6 unit cells, thus a 2DEG is formed at the interface. A series of fringes can be observed at tens of micrometers away from the edge, suggesting that long-propagating SPhPs can exist in the presence of LAO and 2DEG.

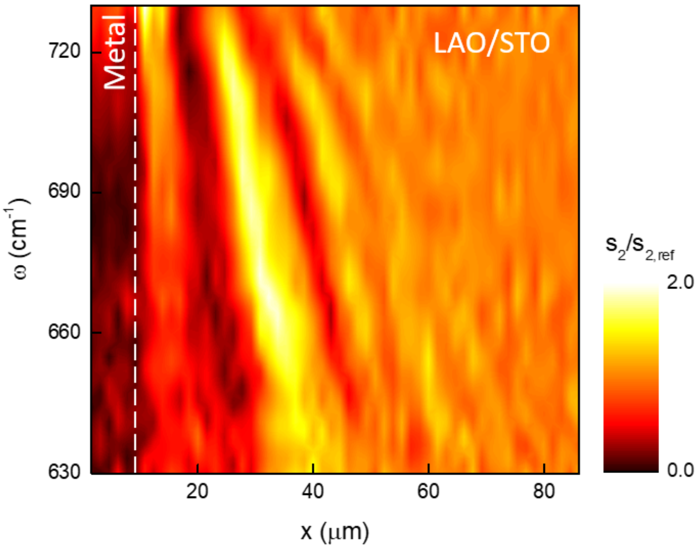

Figure S5. Real-space nano-spectroscopy of SPhPs in LAO/STO at 10 K. Nano-FTIR line scan showing the near-field amplitude $s_2/s_{2,\mathrm{ref}}$ normalized to the signal far from the edge, as a function of the distance x between tip and metal.

S6. Influence of LAO layer on the upper SPhP frequency

In the main text, we discussed that the blueshift of the ω_{onset} for LAO/STO spectra is mainly caused by the 2DEG. Here we demonstrate that this effect cannot be caused by the temperature dependence of the dielectric function of LAO. Figure S6 shows two s-SNOM amplitude spectra of the LAO/STO layered system (without 2DEG for simplicity), simulated by the finite-dipole model, where we used the same dielectric function of STO and two different dielectric functions of LAO (at 10 and 300 K). We can see that the spectra barely shift when only the properties of LAO are changed. The calculation of ω_{onset}, using the same ad hoc method as in the main text gives the values of 733.2 ± 0.05 cm^{-1} and 733.0 ± 0.05 cm^{-1} respectively. The difference of 0.2 cm^{-1} is negligible compared to the shift of about 14 cm^{-1} that we observed (Fig. 3d).

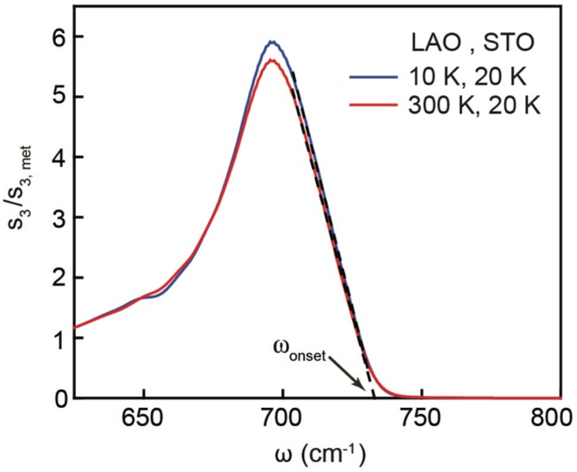

Figure S6. Simulated s-SNOM amplitude spectra of the layered system without 2DEG, obtained using the finite-dipole model. Blue curve: LAO at 10 K and STO at 20 K. Red curve: LAO at 300 K

and STO at 20 K. Dashed lines are the linear fits to the high-frequency slope of the SPhP peak used to determine the onset frequency ω_{onset}.

S7. Correspondence between electronic bands in Nb-STO and LAO/STO based 2DEG

The conduction bands immediately above the gap in $SrTiO_3$ are of Ti d_{xy}, d_{yz}, and d_{zx} character. The d_{xy} orbital disperses strongly along k_x and k_y, and weakly along k_z, and likewise for d_{yz}, and d_{zx}. The result is a set of three quasi-two dimensional bands dispersing in mutually orthogonal planes.[7] Two-dimensional confinement to the *xy* plane in the 2DEG singles out the d_{xy} band, which splits in a series of subbands characterized by the wave-vector of the standing wave along the *z*-axis[8] . Consequently, the band-dispersion in 2D interfaces and in 3D bulk is almost the same, the main difference being that in 3D bulk material the electrons are doped in 3 degenerate quasi-2D bands, whereas in the interfaces only one of these bands is singled out due to the geometrical confinement.

S8. Extraction of the gate voltage-dependent onset frequency of SPhP band

Figure S7 presents the s-SNOM amplitude spectra of LAO/STO (normalized to the peak maximum) collected on a same point of the sample far away from the metal edge at all gate voltages that we applied. The extracted corresponding ω_{onset} as a function of the gate voltages are plotted in Fig. 4c of the main text. The ω_{onset} exhibits a redshift of about 5 cm^{-1} as the voltage is swept from 0 to -200 V, but it almost unvaried due to a low gate efficiency at positive voltages.

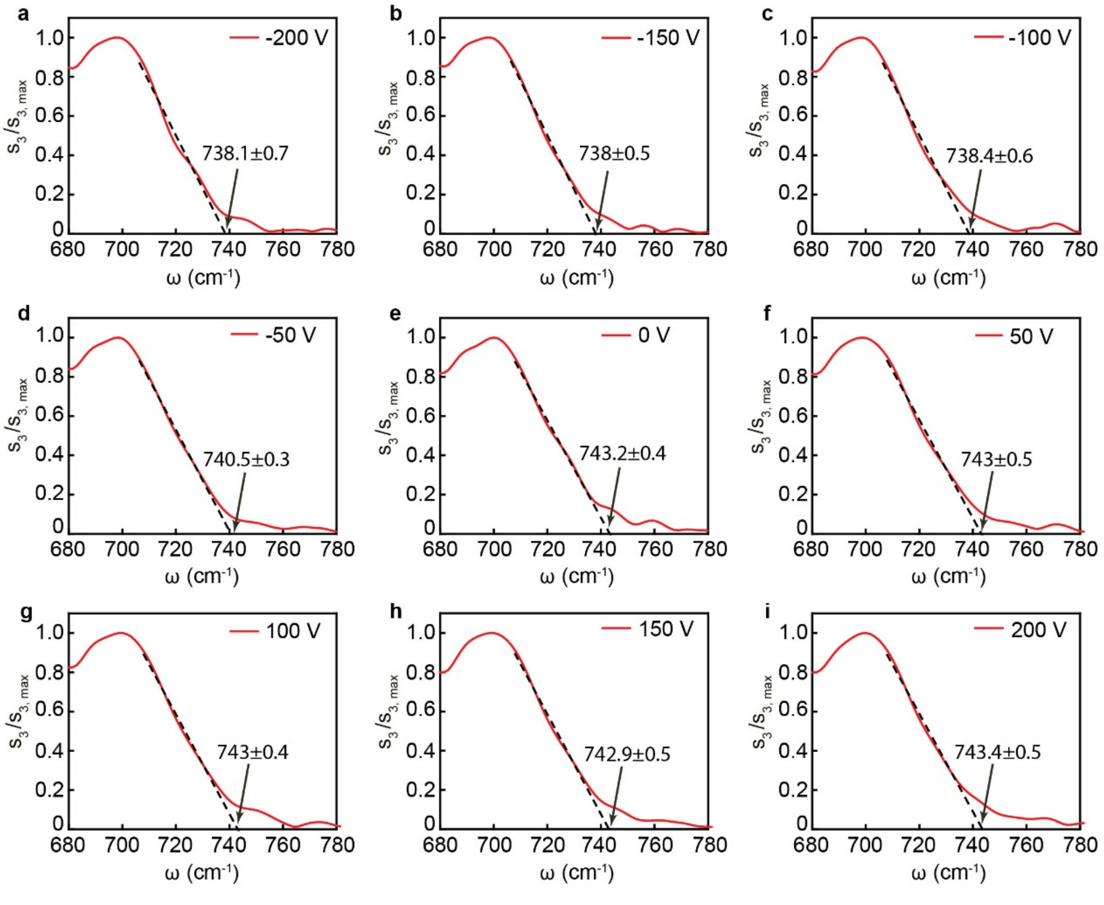

Figure S7. Extraction of the gate-voltage dependent onset frequency of SPhP band for LAO/STO. a-i, Nano-FTIR spectra $s_3/s_{3,\max}$ measured far away from the metal edge at different gate voltages

(7.7 K). Black dashed lines: the linear fits of the data with a high slope, used to obtain the onset frequency ω_{onset} at every gate voltage value (indicated by black arrows).

S9. Hyperspectral maps in LAO/STO samples at different gate voltages

We did a series of the SPhP interferometry measurements on LAO/STO for different gate voltages and the dependence. In Fig. S8 a-c we show three (ω,x) maps taken at V_G = -150V, 0 V and + 150 V. The difference between the spatial signal profiles is small, as one can also see in Fig. S9d.

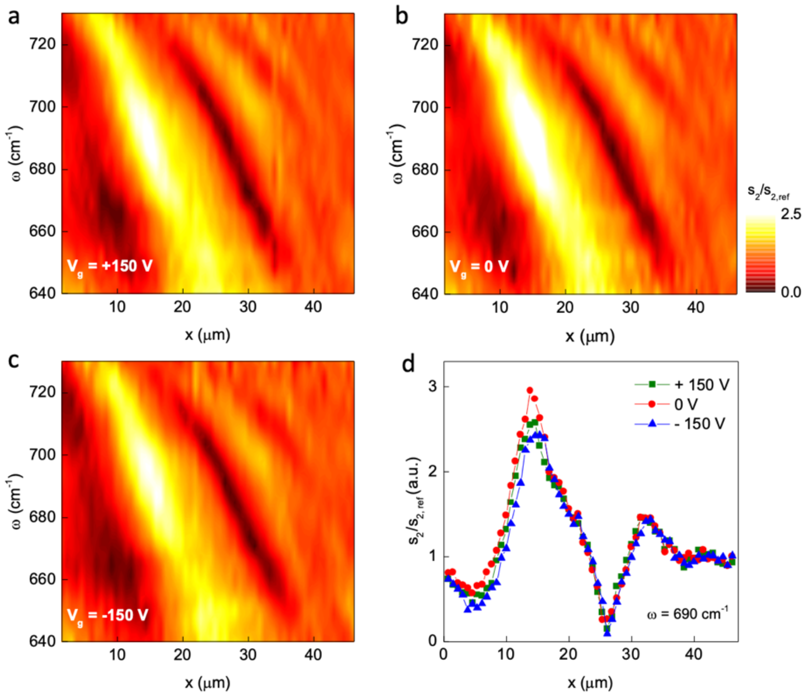

Figure S8. Real-space nano-spectroscopy of SPhPs in LAO/STO at different gate voltages. a-c, Hyperspectral (ω-x) maps of the nano-FTIR amplitude (normalized to signal far from the edge) on LAO/STO at 10 K at V_G = +150 V, 0 V and -150 V respectively. **d**, The spatial profiles at different voltages at ω = 690 cm^{-1}.

S10. Calculated surface-polariton dispersions in the LAO/2DEG/STO system

In Fig. S9 we present calculated maps of $\mathrm{Im}[r_p(q,\omega)]$ of the LAO/2DEG/STO system, which illustrate the dispersion of surface-polaritons in this heterostructure. In Fig. S10a, the calculation is done for the same configuration as for the case shown in Fig. 4d of the main text, for $\delta\varepsilon_1 = 0$, $\delta\varepsilon_2 = 0$. Namely, we assume that at zero gate voltage $\varepsilon_{2DEG}(\omega)$ is equal to $\varepsilon_{STNO}(\omega)$. Two surface polariton branches are observed: the SPhPs (solid line) and the 2DEG plasmon-polaritons[6] (dashed line). In Fig. S10b, we present the same calculation, where the real part of $\varepsilon_{2DEG}(\omega)$ was incremented by +24, which is the simple model mimicking the application of the gate voltage V_G = -150 V in the main text (Fig. 4d). In Fig. S10c we compare both surface-polariton branches for the two cases. One can see that: (i) the low-momentum SPhPs (A) are almost not affected, (ii) the high-momentum SPhPs (B) show a red shift of about 5 cm^{-1} and (iii) the 2DEG surface-polariton branch is strongly modified by the gate (C), in agreement with Ref. [6].

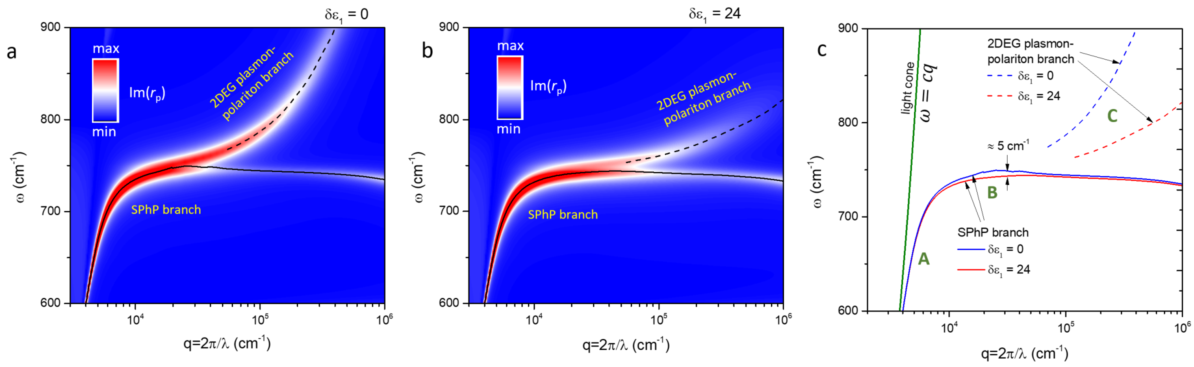

Figure S9. Calculated surface-polariton dispersions in the LAO/2DEG/STO system. a and **b,** Simulated SPhP – plasmon polariton dispersion in the LAO/2DEG/STO system at two gate voltages: 0 (**a**) and -150 V (**b**). Applying voltage is mimicked by adding +24 to the real part of $\varepsilon_{2\mathrm{DEG}}(\omega)$. Solid lines denote the SPhP branch, dashed lines correspond to the 2DEG plasmon polariton branch. The lines are determined as loci of the maxima of $\mathrm{Im}[r_{\mathrm{p}}(\omega)]$ as function of ω for fixed values of q. In panel **c**, the two branches are shown together for the two values of the gate voltage. Region A corresponds to the low-momentum SPhPs, which determine the interference patterns in the hyperspectral (ω-x) maps (Fig. S8). Region B corresponds to the high-momentum SPhPs, which define the onset frequency seen in the nano-FTIR spectra. Region C corresponds to the plasmon-polariton branch, studied in Ref.[6].

Supplementary References

1. Hu F, *et al.* Imaging exciton–polariton transport in MoSe 2 waveguides. *Nature Photonics* **11**, 356-360 (2017).

2. Hu D, *et al.* Probing optical anisotropy of nanometer-thin van der waals microcrystals by near-field imaging. *Nature communications* **8**, 1-8 (2017).

3. Huber A, Ocelic N, Kazantsev D, Hillenbrand R. Near-field imaging of mid-infrared surface phonon polariton propagation. *Applied physics letters* **87**, 081103 (2005).

4. Cvitkovic A, Ocelic N, Hillenbrand R. Analytical model for quantitative prediction of material contrasts in scattering-type near-field optical microscopy. *Opt Express* **15**, 8550-8565 (2007).

5. Hauer B, Engelhardt AP, Taubner T. Quasi-analytical model for scattering infrared near-field microscopy on layered systems. *Opt Express* **20**, 13173-13188 (2012).

6. Luo W, *et al.* High sensitivity variable-temperature infrared nanoscopy of conducting oxide interfaces. *Nature communications* **10**, 1-8 (2019).

7. Van Der Marel D, van Mechelen JLM, Mazin I. Common Fermi-liquid origin of T 2 resistivity and superconductivity in n-type SrTiO 3. *Physical Review B* **84**, 205111 (2011).

8. Delugas P, Filippetti A, Fiorentini V, Bilc DI, Fontaine D, Ghosez P. Spontaneous 2-dimensional carrier confinement at the n-type SrTiO 3/LaAlO 3 interface. *Physical review letters* **106**, 166807 (2011).
